# Successful Prevention of Bronchopleural Fistula in Single-Stage Esophagectomy and Right Lower Lobectomy: A Case Report

**DOI:** 10.70352/scrj.cr.25-0170

**Published:** 2025-06-11

**Authors:** Tomonari Oki, Shuhei Iizuka, Makoto Tomatsu, Toru Nakamura

**Affiliations:** 1 Department of General Thoracic Surgery, Seirei Hamamatsu General Hospital, Hamamatsu, Shizuoka, Japan; 2 Department of Gastrointestinal Surgery, Seirei Hamamatsu General Hospital, Hamamatsu, Shizuoka, Japan

**Keywords:** esophagectomy, lobectomy, bronchial fistula, bronchial arteries, intercostal muscles

## Abstract

**INTRODUCTION:**

Bronchopleural fistulae (BPFs) following pulmonary resection are potentially fatal complications, with right lower lobectomy being the most susceptible among lobectomies. As esophagectomy also increases the risk of tracheobronchial ischemia and postoperative malnutrition, performing a single-stage esophagectomy combined with right lower lobectomy may further elevate the risk of BPFs, underscoring the need for meticulous preoperative planning.

**CASE PRESENTATION:**

A 64-year-old male with a history of heavy smoking was referred to our hospital after an abnormal mass was detected on a chest radiograph during an annual health check. Chest CT revealed a 3.7 cm consolidative mass in the right lower lobe, resulting in a diagnosis of primary lung cancer, classified as T2aN0M0, stage IB. Additionally, abnormal fluorodeoxyglucose (FDG) uptake was observed in the lower thoracic esophagus, leading to a diagnosis of synchronous esophageal cancer, classified as T1bN0M0, stage I. As both lesions required upfront surgical resection via the right thoracic cavity, a single-stage esophagectomy and right lower lobectomy were planned. Initially, esophagectomy was performed using a five-port video-assisted thoracic surgery (VATS) approach in the prone position from the right side. To preserve the blood supply to the fifth intercostal muscle for subsequent harvesting as a muscle flap, the utility port in the corresponding intercostal space was placed as ventrally as possible. The esophagectomy was performed while preserving the right main bronchial artery. The patient was then repositioned to the left lateral decubitus position, and the preserved fifth intercostal muscle flap was harvested. A right lower lobectomy was completed, preserving the bronchial artery, and the bronchial stump was reinforced using the harvested muscle flap. Despite postoperative development of esophagogastric anastomotic leakage, the patient did not develop a BPF, and no signs of BPF have been observed during 12 months of follow-up.

**CONCLUSIONS:**

Preservation of the right main bronchial artery and reinforcement of the bronchial stump with an intercostal muscle flap facilitated prevention of BPF following single-stage esophagectomy and right lower lobectomy, despite the patient’s history of heavy smoking and transient postoperative malnutrition.

## Abbreviations

BMI
: body mass index
BPF
: bronchopleural fistula
FDG
: fluorodeoxyglucose
PNI
: prognostic nutritional index
VATS
: video-assisted thoracic surgery

## INTRODUCTION

BPFs following pulmonary resection for lung cancer are potentially fatal complications. A history of heavy smoking, bronchial ischemia, malnutrition, and a right lower lobectomy are identified risk factors for BPFs. As esophagectomy also increases the risk of tracheobronchial ischemia and postoperative malnutrition, performing a single-stage esophagectomy combined with right lower lobectomy may further elevate the risk of BPFs, underscoring the need for meticulous preoperative planning. We herein report a successful case of preventing a postoperative BPF by preserving the right bronchial artery and reinforcing the bronchial stump with an intercostal muscle flap in a heavy smoker with synchronous primary lung cancer of the right lower lobe and thoracic esophageal cancer.

## CASE PRESENTATION

**Fig. 1:**
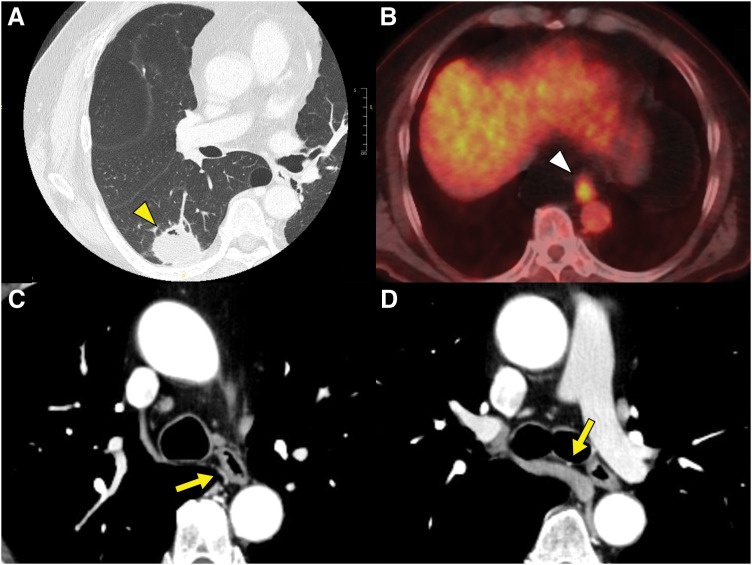
Chest CT revealed a 3.7 cm consolidative mass with irregular margins in the right lower lobe (yellow arrowhead in **A**). PET demonstrated an abnormal FDG uptake in the lower thoracic esophagus (white arrowhead in **B**). The right bronchial artery (yellow arrows) originated from the descending aorta, traversed dorsally to the esophagus (**C**), and passed beneath the azygos arch to supply the right bronchus (**D**).

A 64-year-old male was referred to our hospital for an abnormal mass detected in the right lower lung field on a chest radiograph during an annual health check. He had maintained a daily smoking habit of 20 cigarettes for 44 years, beginning at the age of 20 and continuing up to the time of surgery, resulting in a Brinkman Index of 880. Chest CT revealed a 3.7 cm consolidative mass in the right lower lobe (**Fig. 1A**). Bronchoscopy identified a squamous cell carcinoma, resulting in a diagnosis of primary lung cancer, classified as T2aN0M0, stage IB. Additionally, abnormal FDG uptake was observed in the lower thoracic esophagus (**Fig. 1B**), and a quarter circumference 0-IIc lesion was identified on upper gastrointestinal endoscopy. Biopsy confirmed an adenocarcinoma leading to a diagnosis of synchronous esophageal cancer, classified as T1bN0M0, stage I. As both lesions required upfront surgical resection via the right thoracic cavity, a single-stage esophagectomy and right lower lobectomy were planned to avoid the technical difficulties associated with adhesions in a two-stage procedure.

Due to the anticipated risk of a BPF resulting from reduced blood supply to the right bronchus and malnutrition following the esophagectomy, we underscored the importance of preserving the blood flow to the right bronchus during the esophagectomy and reinforcing the bronchial stump after the lobectomy. The chest CT revealed that the right bronchial artery originated from the descending aorta, traversed dorsally to the esophagus (**Fig. 1C**), and passed beneath the azygos arch (**Fig. 1D**) to supply the right bronchus.

**Fig. 2:**
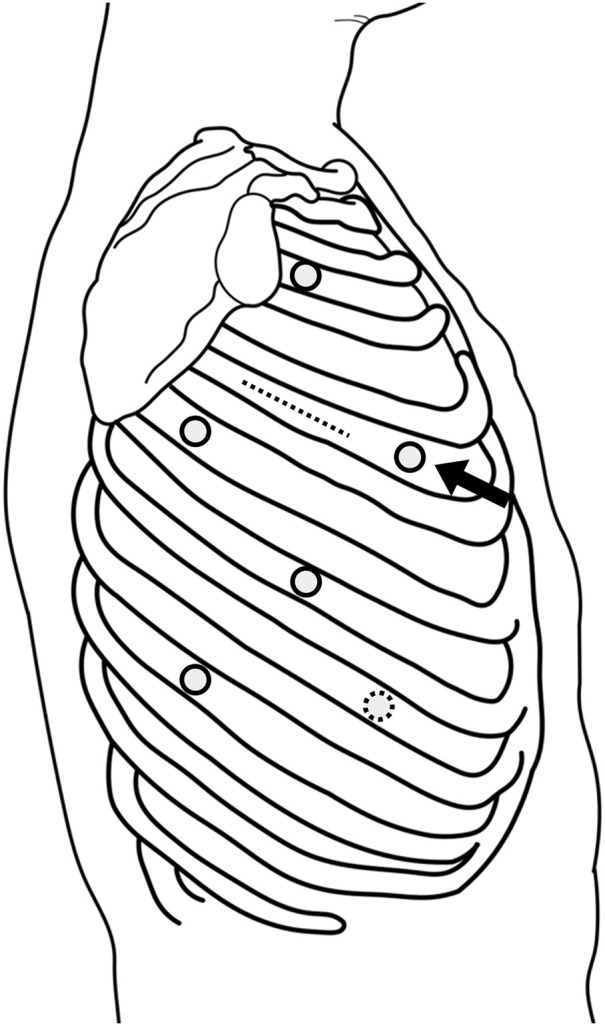
Esophagectomy was performed via 5-mm ports at the posterior axillary line of the third intercostal space, the midaxillary line of the fifth intercostal space, and the subscapular line of the sixth intercostal space, as well as 12-mm ports at the posterior axillary line of the seventh intercostal space and the subscapular line of the ninth intercostal space (solid circles). To preserve the fifth intercostal muscle for subsequent harvesting as a muscle flap, the port in the corresponding intercostal space was placed as ventrally as possible (black arrow). The intercostal muscle flap was harvested and a right lower lobectomy was completed via an 8-cm mini-thoracotomy at the posterior axillary line of the fifth intercostal space (dotted line), utilizing a 12-mm port at the midaxillary line of the eighth intercostal space (dotted circle). Separate ports were placed for the esophagectomy and pulmonary resection, with no shared ports.

**Fig. 3:**
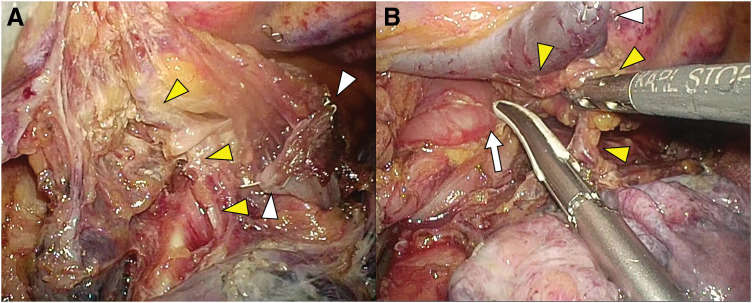
The full-length of the right bronchial artery (yellow arrowheads) was identified by transecting the azygos arch (white arrowheads in **A**). The esophagus (white arrow in **B**) was carefully dissected, preserving the bronchial artery (yellow arrowheads).

**Fig. 4:**
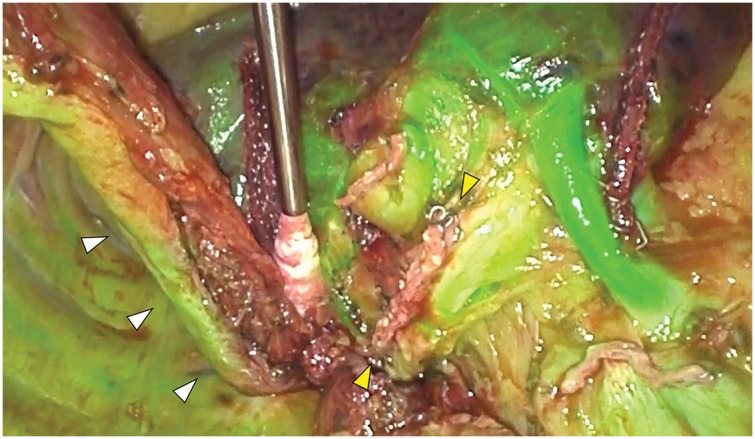
After a right lower lobectomy, sufficient blood supply to the bronchial stump (yellow arrowheads) and the harvested intercostal muscle flap (white arrowheads) was confirmed by fluorescence imaging.

Initially, esophagectomy was performed using a five-port VATS approach in the prone position from the right side (**Fig. 2**). To preserve the blood supply to the fifth intercostal muscle for subsequent harvesting as a muscle flap during the right lower lobectomy, the utility port in the corresponding intercostal space was placed as ventrally as possible (**Fig. 2**). The azygos arch was transected (**Fig. 3A**), and the esophagus, along with the subcarinal lymph nodes, were carefully dissected from the bronchial artery. After a cranial transection of the esophagus, it was mobilized caudally, passing beneath the bronchial artery to facilitate esophagectomy (**Fig. 3B**). The patient was then repositioned to the left lateral decubitus position, and the preserved fifth intercostal muscle flap was harvested via a mini-thoracotomy at the fifth intercostal space (**Fig. 2**). A right lower lobectomy was completed via the incision, preserving the entire bronchial artery. Intravenous indocyanine green (12.5 mg) was administered, and fluorescence imaging confirmed a sufficient blood supply to the bronchial stump and the harvested intercostal muscle flap (**Fig. 4**). The bronchial stump was reinforced using the harvested muscle flap, which was sutured with 4-0 monofilaments. The patient was then repositioned to the supine position and the esophagectomy with a gastric conduit reconstruction via the retrosternal route was completed. The operative time was 238 minutes for esophagectomy, 269 minutes for right lower lobectomy, and 411 minutes for gastric conduit reconstruction, with a total estimated blood loss of 85 mL. Due to obesity, with a BMI of 31.2, the abdominal procedures required a prolonged operative time.

A pathological examination revealed esophageal cancer classified as T1bN0M0, stage I, consistent with the preoperative staging. The lung cancer, measuring 4.1 cm, was diagnosed as T2bN0M0, stage IIA. The postoperative course following the lobectomy was uneventful, and the thoracic drain was removed on the fifth postoperative day. However, the patient required parenteral nutrition during the following 18 days due to esophagogastric anastomotic leakage and was discharged home 34 days after surgery. His preoperative BMI was 31.2, with a serum albumin level of 4.2 g/dL and a PNI of 57.4; however, due to malnutrition induced by parenteral nutrition and inflammation resulting from an anastomotic leak, the serum albumin level declined to 2.1 g/dL, and the PNI decreased to 25.5 by postoperative day 8. Following the resumption of oral intake, the patient’s nutritional status gradually improved, with serum albumin levels recovering to 3.3 g/dL and the PNI reaching 45.6 at the time of discharge. Although his body weight continued to decline post-discharge due to the sequelae of esophagectomy, with the BMI decreasing to 25.7 over 12 months follow-up, he did not develop a BPF.

## DISCUSSION

A BPF following pulmonary resection for lung cancer is among the most critical postoperative complications, with an incidence reported between 0.44% and 1.2% and a mortality rate ranging from 3.8% to 14%.^1,2)^ Additional risk factors for a BPF include a history of heavy smoking, right-sided surgery, bronchial stump ischemia, diabetes mellitus, and hypoalbuminemia.^2–4)^

Postoperative bronchial ischemia is a primary etiological factor for BPF and predominantly affects the right intermediate bronchial trunk.^4)^ Consequently, right lower lobectomy is the most prone to developing BPF among single lobectomies, following a right pneumonectomy and right middle and lower bilobectomy.^2,3)^ Preservation of the bronchial artery is crucial for preventing postoperative bronchial ischemia. The right bronchial blood flow is supplied by the right main bronchial artery, which originates from the descending aorta and subsequently joins branches from the right third intercostal artery, passing beneath the azygos arch.^5)^ These anatomical characteristics pose a potential risk of incidental bronchial artery injury during esophageal dissection and lymphadenectomy in the posterior mediastinum.^6)^ Therefore, preservation of bronchial blood flow and careful esophageal dissection are crucial for preventing postoperative bronchial ischemia.^7)^

Nutrition plays a pivotal role in wound healing, as nutritional deficiencies impede this process by hindering fibroblast proliferation, collagen synthesis, epithelialization, and other essential mechanisms.^8)^ After esophagectomy, nutritional status may be impaired for several months to years, with the possibility of never fully returning to preoperative levels.^9)^ Therefore, simultaneous esophagectomy and right lower lobectomy pose an exceedingly high risk of BPF due to vascular impairment and postoperative malnutrition, both of which hinder bronchial stump healing.

As a preventive measure for BPF following a pulmonary resection, reinforcement of the bronchial stump with autologous tissue is a feasible option. Tissues such as intercostal muscle, parietal pleura, pericardial fat, and greater omentum have been employed.^10)^ The intercostal muscle flap is a highly vascularized tissue that can be harvested easily and safely,^11,12)^ and its efficacy in the prevention of BPF has been well-documented.^13–15)^ After right lower lobectomy, the bronchial stump is exposed to the free space within the thoracic cavity; thus, establishing a closed environment around the bronchial stump may facilitate wound healing.^16)^ Furthermore, the intercostal muscle flap may also enhance blood supply to the bronchial stump.^17)^

The following precautionary measures were taken to prevent BPF in the present case. The dorsal fifth intercostal space was preserved as much as possible during VATS port placement to facilitate subsequent intercostal muscle harvesting. The right main bronchial artery was completely identified and preserved to maintain bronchial blood flow during esophagectomy. Consequently, sufficient blood flow to the preserved muscle flap and bronchial stump was confirmed by fluorescence imaging. These procedures successfully prevented BPF. Interprofessional collaboration between gastrointestinal and general thoracic surgeons prior to surgery also played an essential role in establishing the preoperative treatment strategy.

## CONCLUSIONS

Preservation of the right main bronchial artery and reinforcement of the bronchial stump with an intercostal muscle flap facilitated prevention of BPF following single-stage esophagectomy and right lower lobectomy, despite the patient’s history of heavy smoking and transient postoperative malnutrition.

## ACKNOWLEDGMENTS

We thank Mr. John Martin for his proofreading of the manuscript.

## DECLARATIONS

### Funding

Not applicable.

### Authors’ contributions

TO drafted the manuscript.

SI and MT contributed to preparing the manuscript.

TN supervised the final manuscript.

All authors participated in the treatment of the patient.

All authors read and approved the final manuscript.

### Availability of data and materials

Data sharing is not applicable to this article as no datasets were generated or analyzed during the current study.

### Ethics approval and consent to participate

The manuscript does not include any identifying information. Our institution does not require ethics approval for case reports.

### Consent for publication

Written informed consent for the publication of this report was obtained from the patient.

### Competing interests

The authors declare that they have no competing interests.
